# Supplementary figures and images for: Espin enhances confined cell migration by promoting filopodia formation and contributes to cancer metastasis
Source: EMBO Rep. 2025 Apr 4;26(10):2574–96. doi: 10.1038/s44319-025-00437-1 (PMC12117036; doi:10.1038/s44319-025-00437-1)

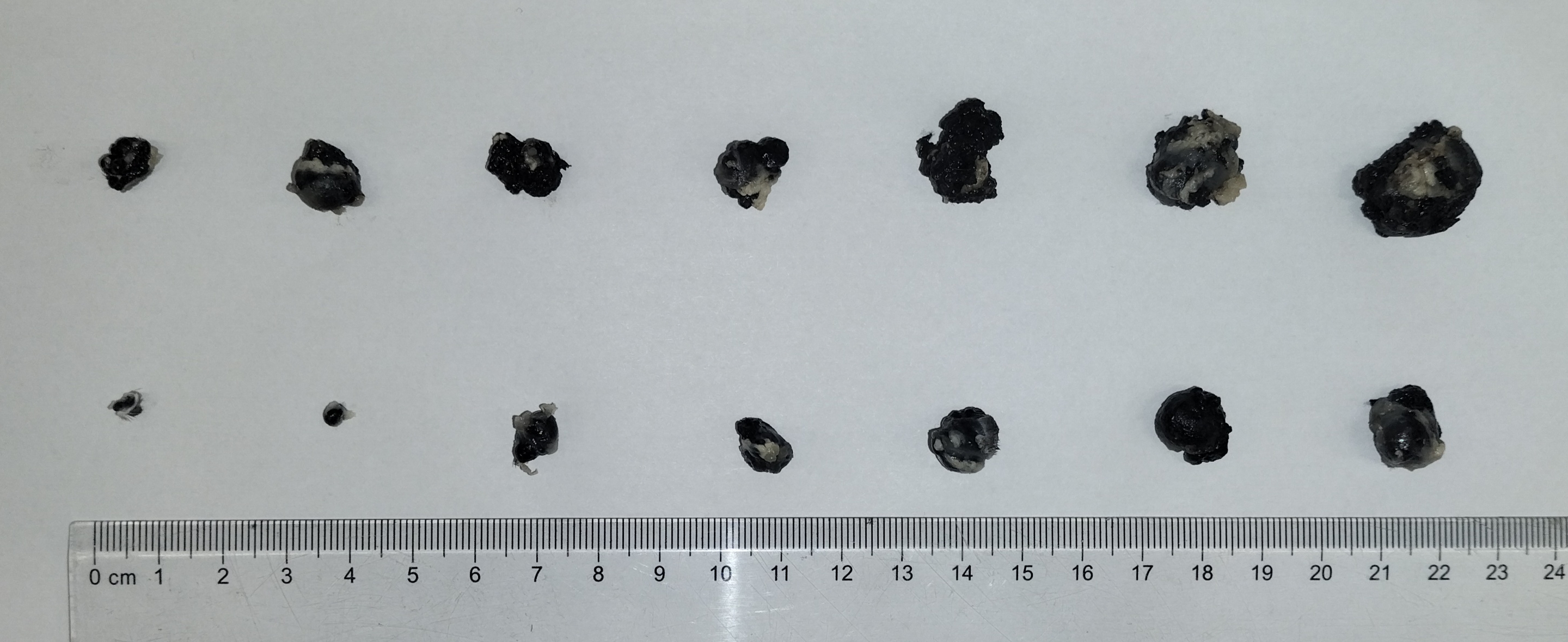

Supplement: Supplementary file 3 — Source data Fig. 2 [file 44319_2025_437_MOESM3_ESM.zip › Figure 2/Figure 2D/Figure 2D.jpg]

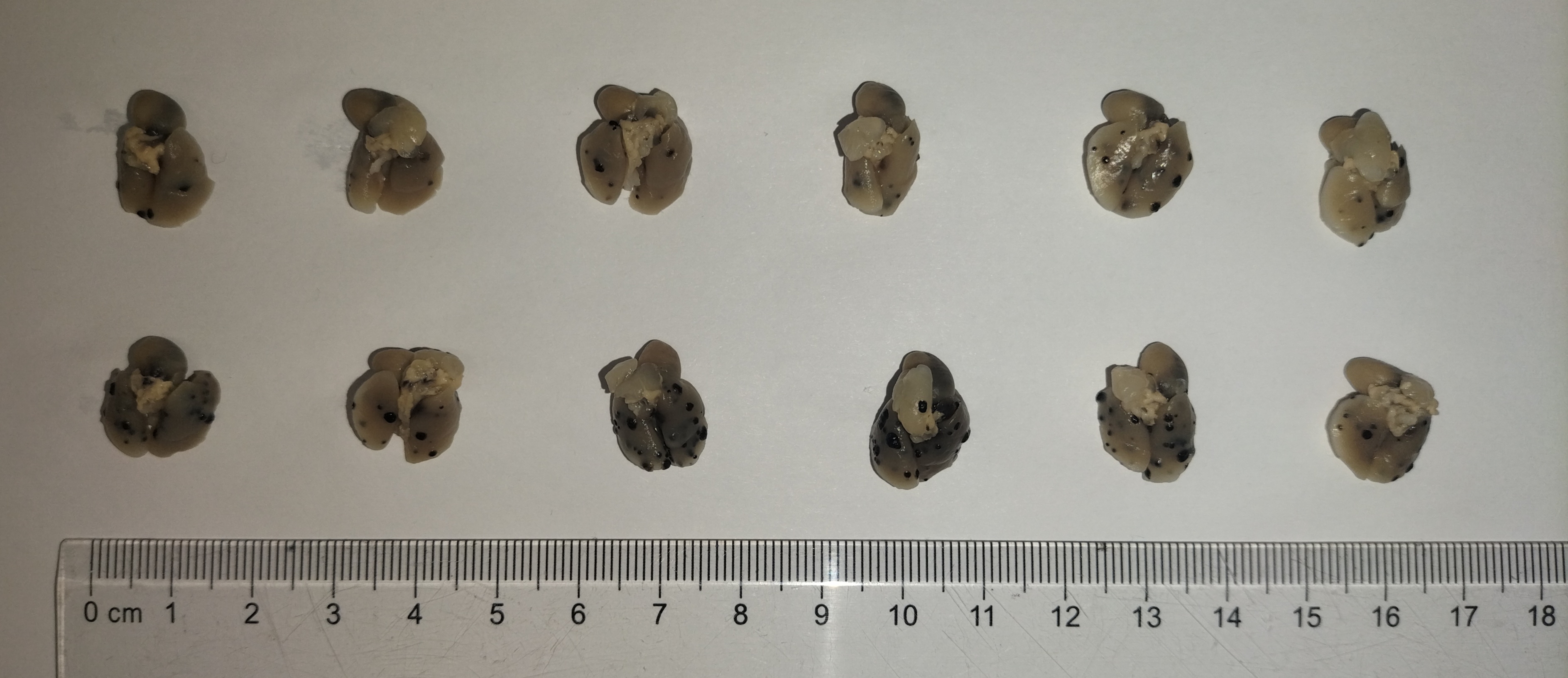

Supplement: Supplementary file 3 — Source data Fig. 2 [file 44319_2025_437_MOESM3_ESM.zip › Figure 2/Figure 2G/Figure 2G.jpg]

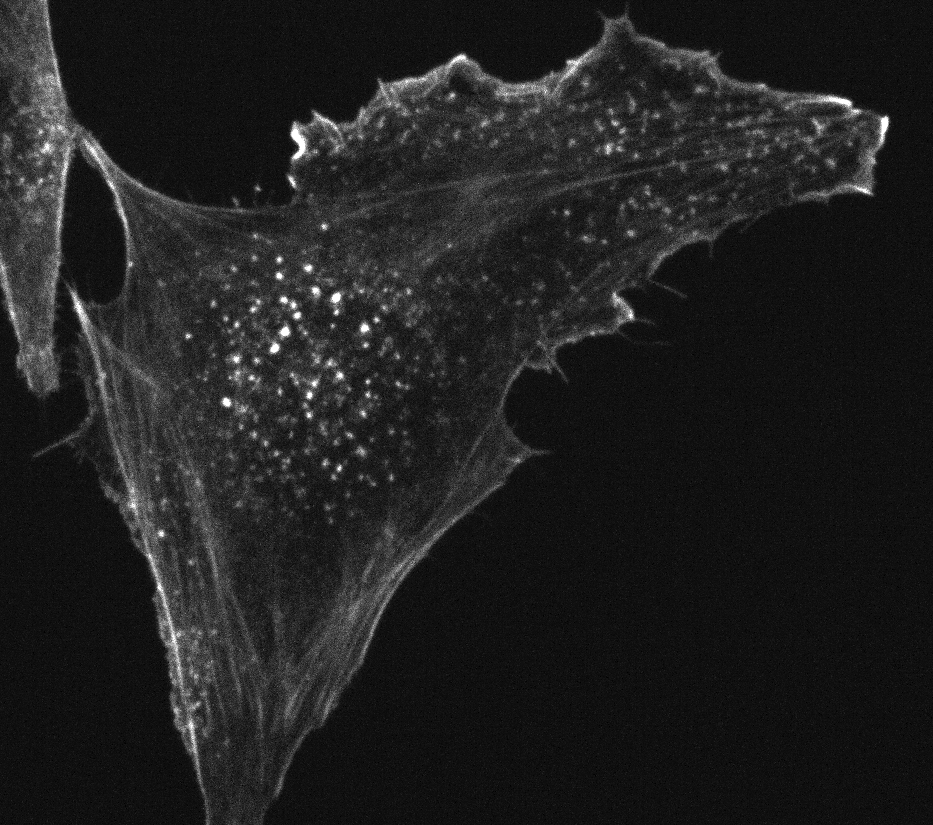

Supplement: Supplementary file 4 — Source data Fig. 3 [file 44319_2025_437_MOESM4_ESM.zip › Figure 3/Figure 3A/Figure 3A.tif]

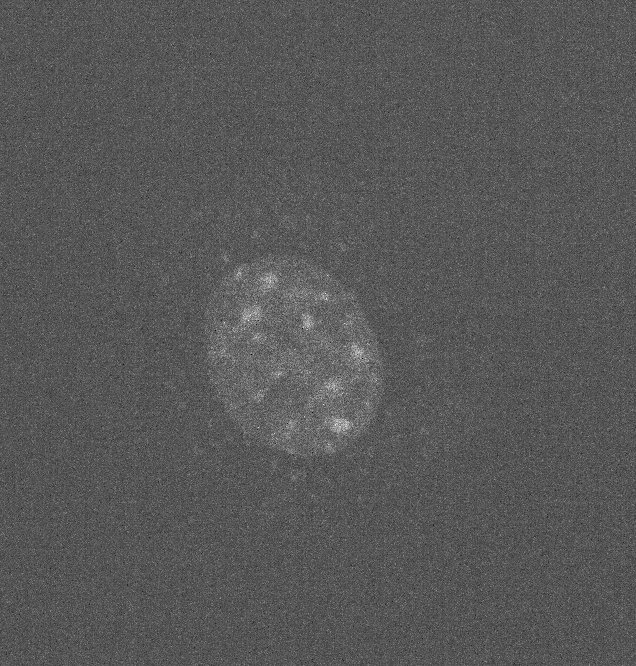

Supplement: Supplementary file 4 — Source data Fig. 3 [file 44319_2025_437_MOESM4_ESM.zip › Figure 3/Figure 3B/Ctrl.tif]

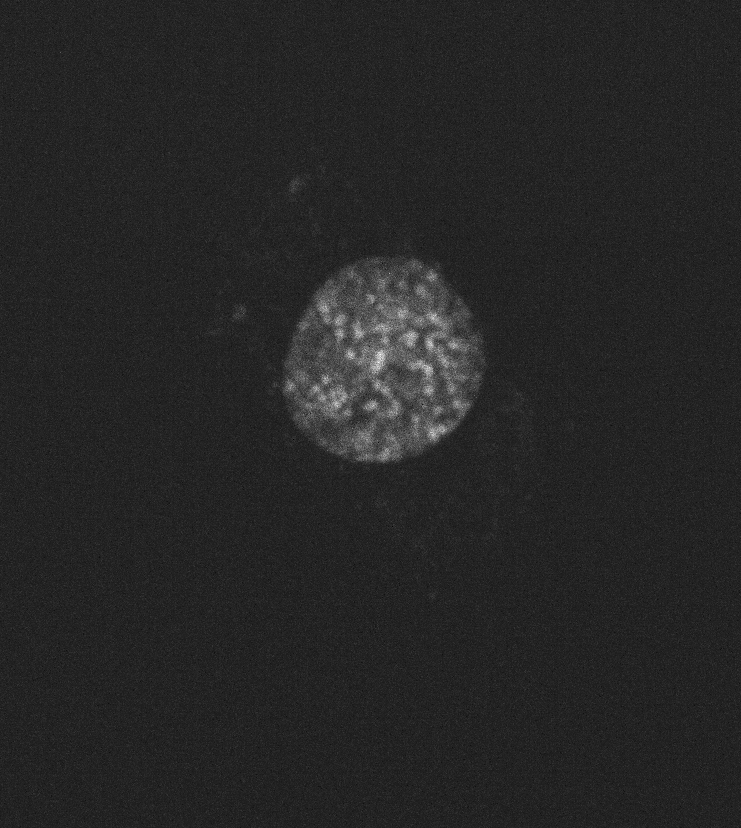

Supplement: Supplementary file 4 — Source data Fig. 3 [file 44319_2025_437_MOESM4_ESM.zip › Figure 3/Figure 3B/Espin OE.tif]

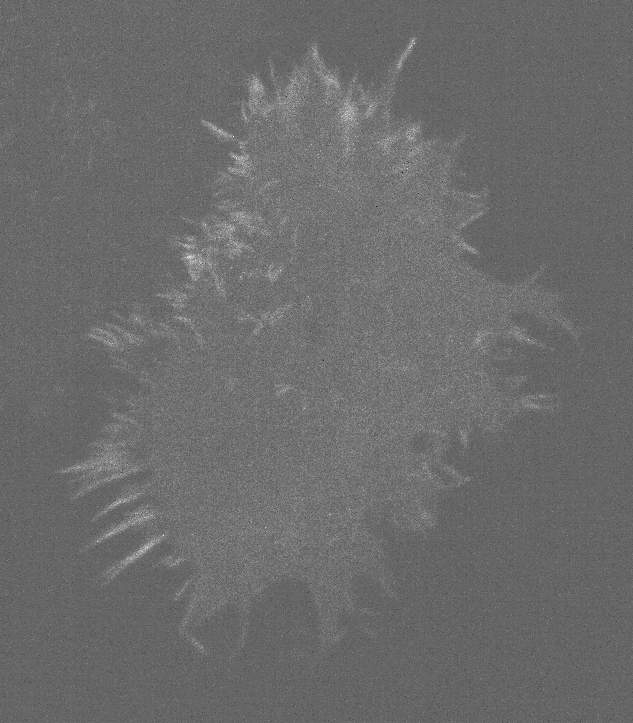

Supplement: Supplementary file 4 — Source data Fig. 3 [file 44319_2025_437_MOESM4_ESM.zip › Figure 3/Figure 3E/Figure 3E.tif]

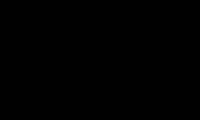

Supplement: Supplementary file 5 — Source data Fig. 4 [file 44319_2025_437_MOESM5_ESM.zip › Figure 4/Figure 4A/Figure 4A.tif]

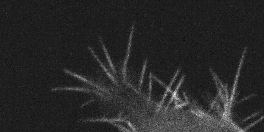

Supplement: Supplementary file 5 — Source data Fig. 4 [file 44319_2025_437_MOESM5_ESM.zip › Figure 4/Figure 4B/Figure 4B.tif]

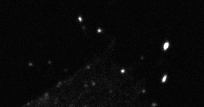

Supplement: Supplementary file 5 — Source data Fig. 4 [file 44319_2025_437_MOESM5_ESM.zip › Figure 4/Figure 4C/Figure 4C.tif]

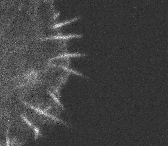

Supplement: Supplementary file 5 — Source data Fig. 4 [file 44319_2025_437_MOESM5_ESM.zip › Figure 4/Figure 4D/Figure 4D.tif]

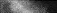

Supplement: Supplementary file 5 — Source data Fig. 4 [file 44319_2025_437_MOESM5_ESM.zip › Figure 4/Figure 4D/kymograph-extending.png]

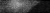

Supplement: Supplementary file 5 — Source data Fig. 4 [file 44319_2025_437_MOESM5_ESM.zip › Figure 4/Figure 4D/kymograph-retracting.png]

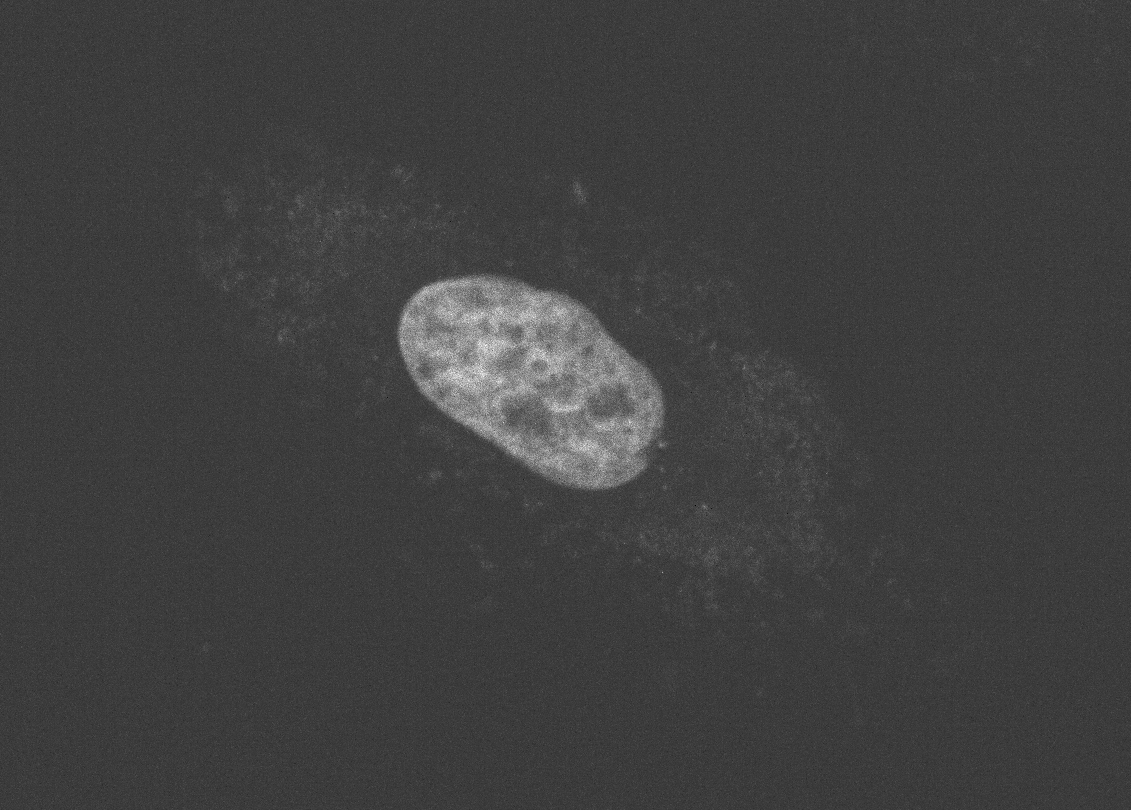

Supplement: Supplementary file 5 — Source data Fig. 4 [file 44319_2025_437_MOESM5_ESM.zip › Figure 4/Figure 4E/Ctrl.tif]

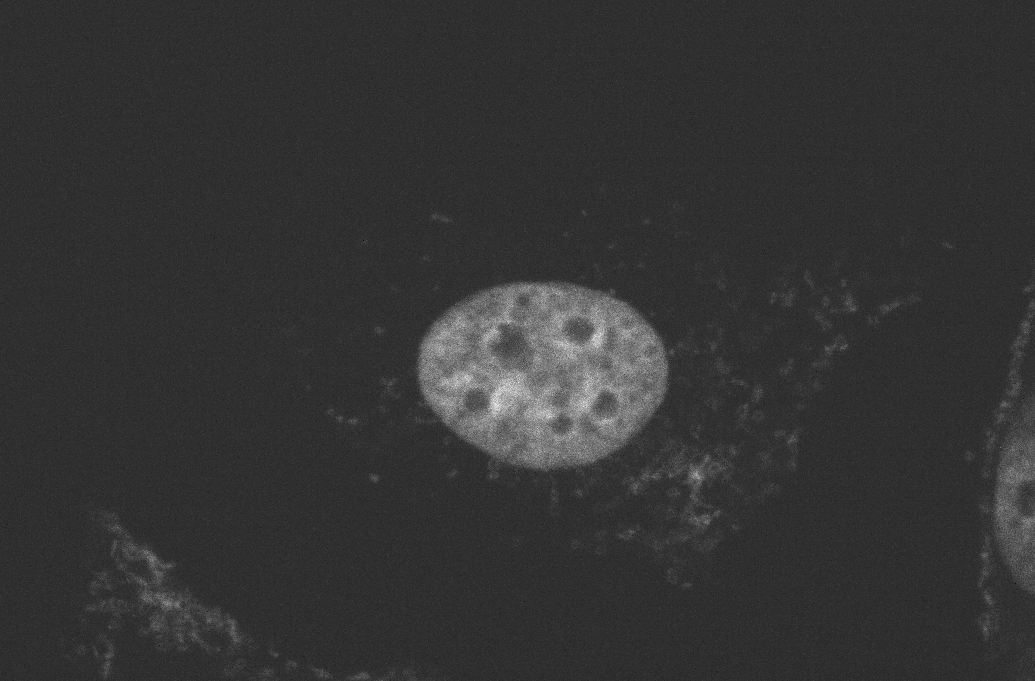

Supplement: Supplementary file 5 — Source data Fig. 4 [file 44319_2025_437_MOESM5_ESM.zip › Figure 4/Figure 4E/Espin OE.tif]

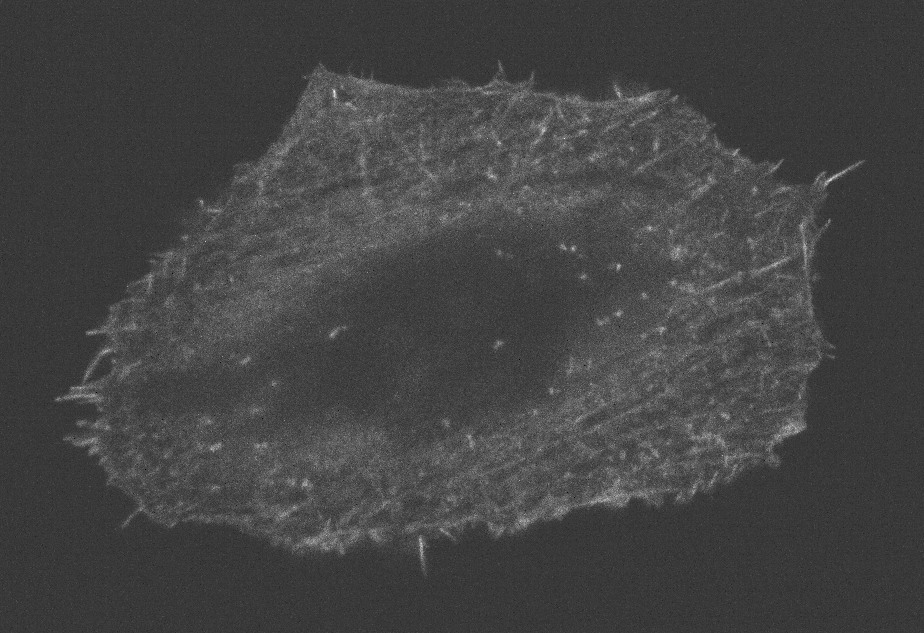

Supplement: Supplementary file 5 — Source data Fig. 4 [file 44319_2025_437_MOESM5_ESM.zip › Figure 4/Figure 4G/CK666.tif]

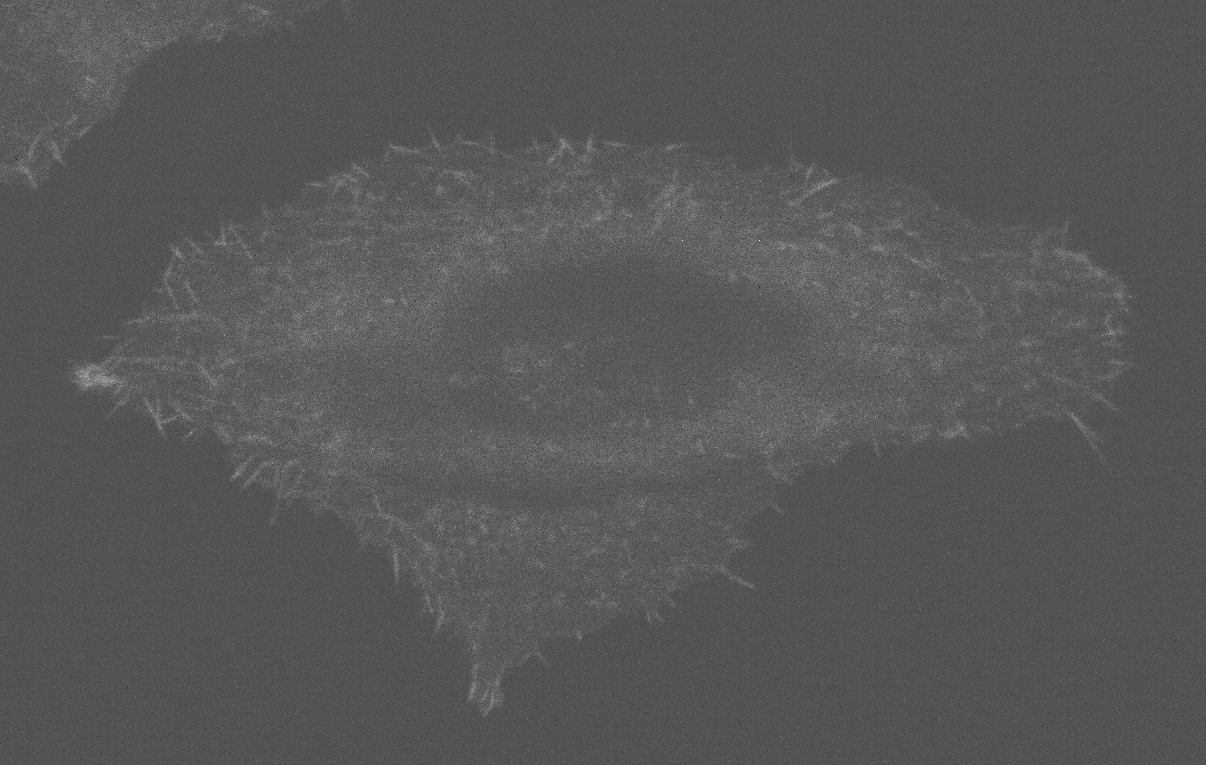

Supplement: Supplementary file 5 — Source data Fig. 4 [file 44319_2025_437_MOESM5_ESM.zip › Figure 4/Figure 4G/DMSO.tif]

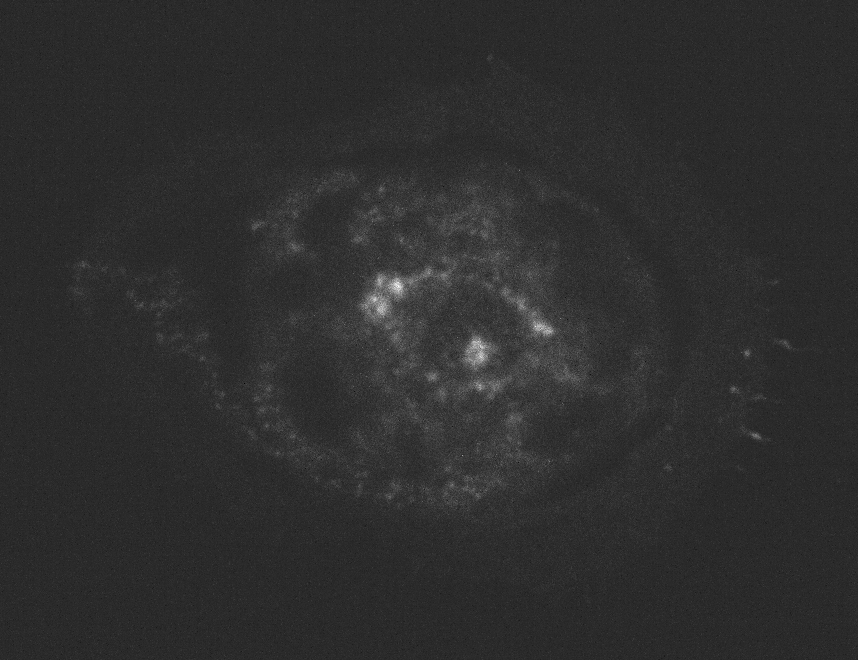

Supplement: Supplementary file 5 — Source data Fig. 4 [file 44319_2025_437_MOESM5_ESM.zip › Figure 4/Figure 4G/SMIFH2.tif]

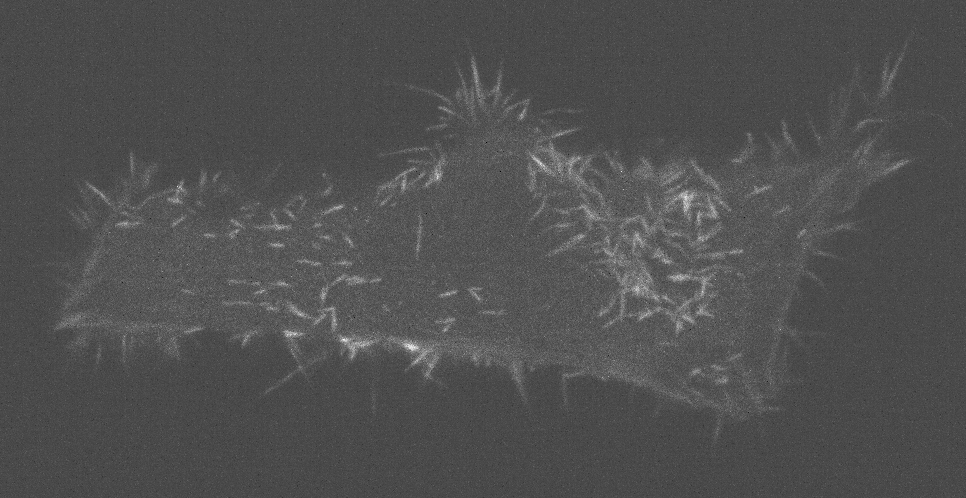

Supplement: Supplementary file 5 — Source data Fig. 4 [file 44319_2025_437_MOESM5_ESM.zip › Figure 4/Figure 4I/Espin OE.tif]

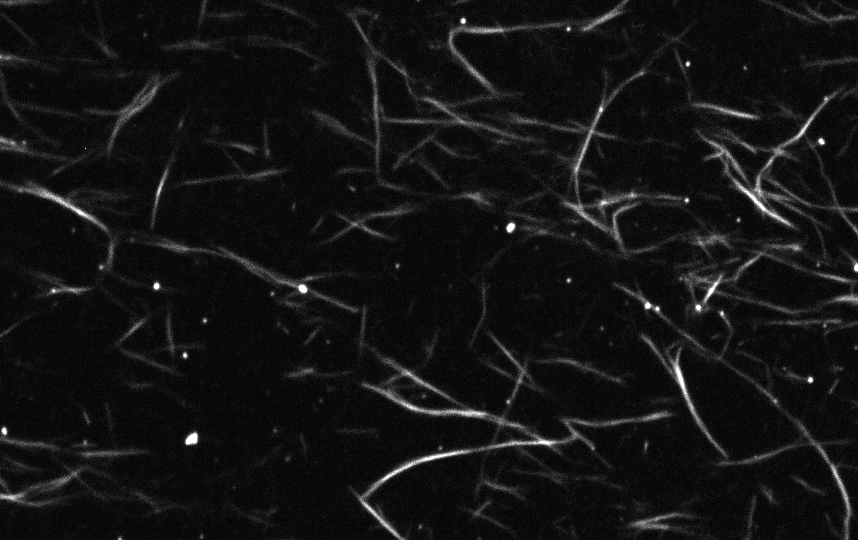

Supplement: Supplementary file 5 — Source data Fig. 4 [file 44319_2025_437_MOESM5_ESM.zip › Figure 4/Figure 4L/+1 μM Espin.tif]

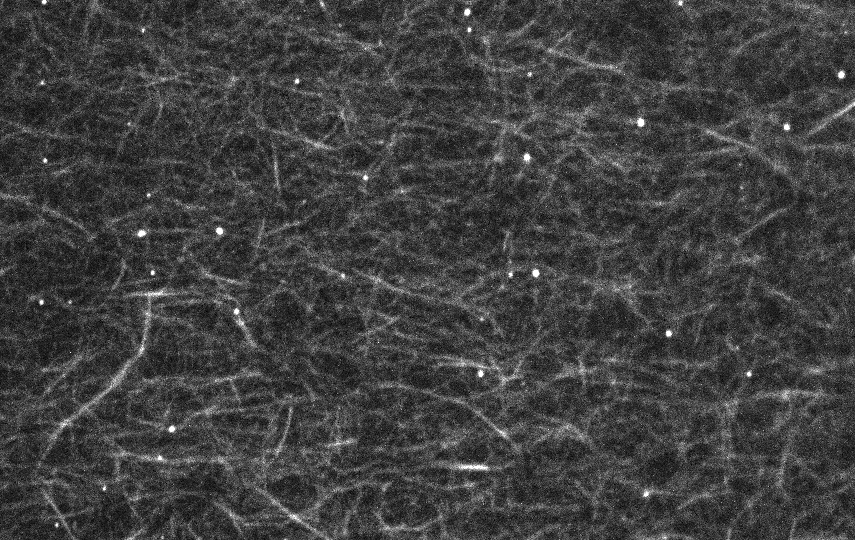

Supplement: Supplementary file 5 — Source data Fig. 4 [file 44319_2025_437_MOESM5_ESM.zip › Figure 4/Figure 4L/+1 μM Fascin.tif]

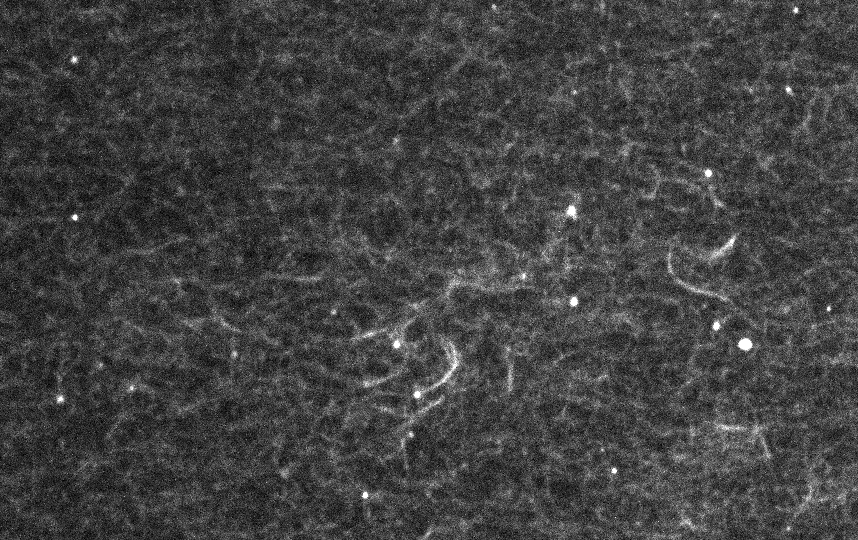

Supplement: Supplementary file 5 — Source data Fig. 4 [file 44319_2025_437_MOESM5_ESM.zip › Figure 4/Figure 4L/+100 nM Espin.tif]

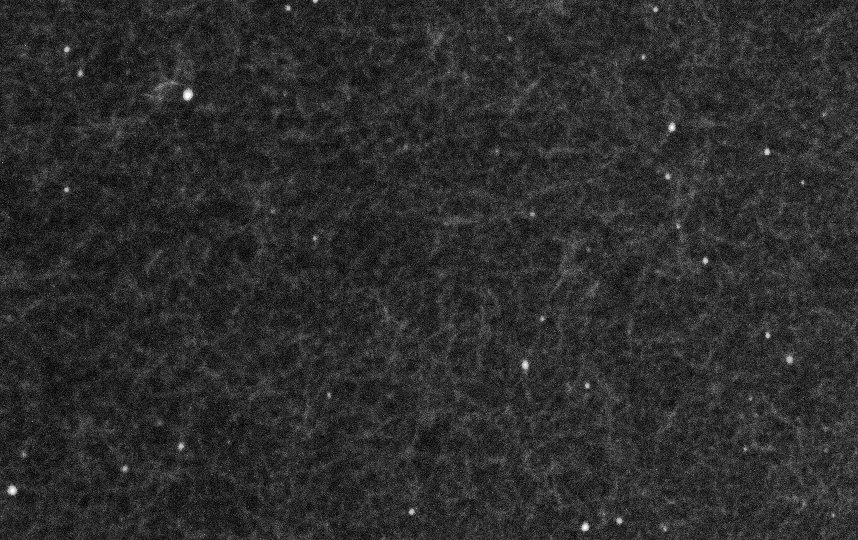

Supplement: Supplementary file 5 — Source data Fig. 4 [file 44319_2025_437_MOESM5_ESM.zip › Figure 4/Figure 4L/+100 nM Fascin.tif]

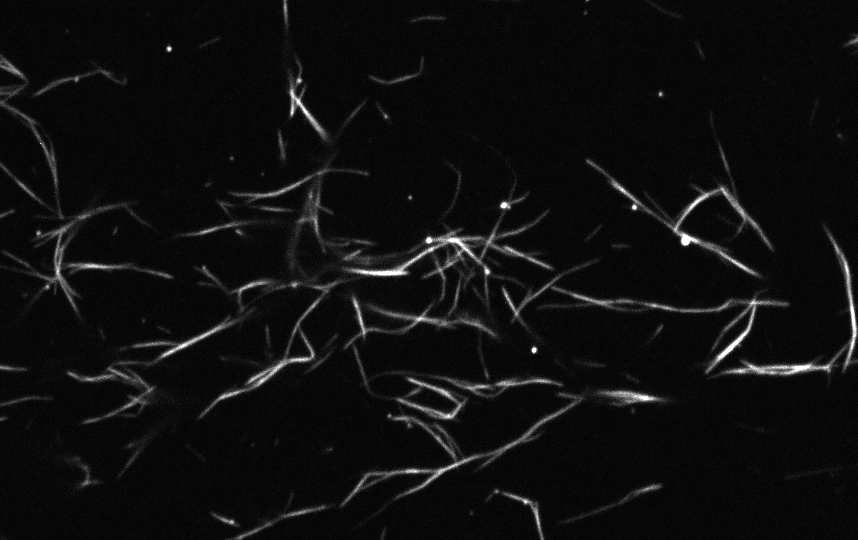

Supplement: Supplementary file 5 — Source data Fig. 4 [file 44319_2025_437_MOESM5_ESM.zip › Figure 4/Figure 4L/+2 μM Espin.tif]

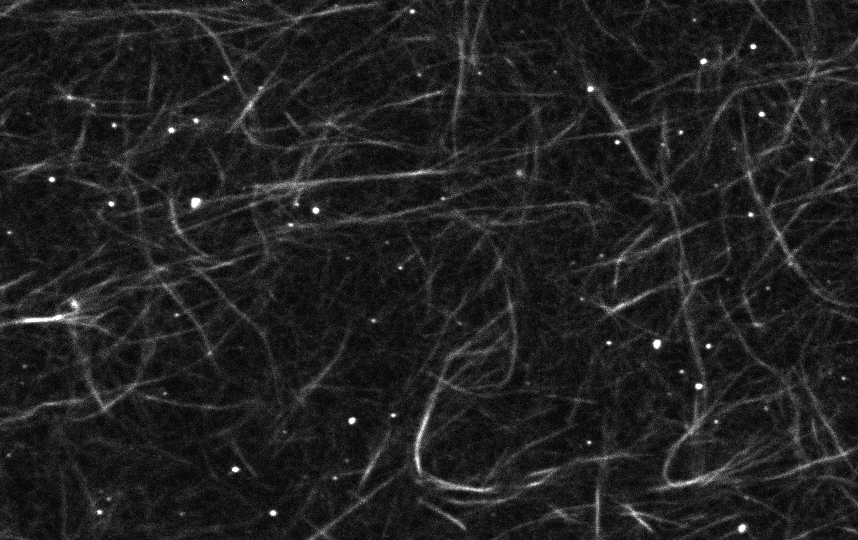

Supplement: Supplementary file 5 — Source data Fig. 4 [file 44319_2025_437_MOESM5_ESM.zip › Figure 4/Figure 4L/+2 μM Fascin.tif]

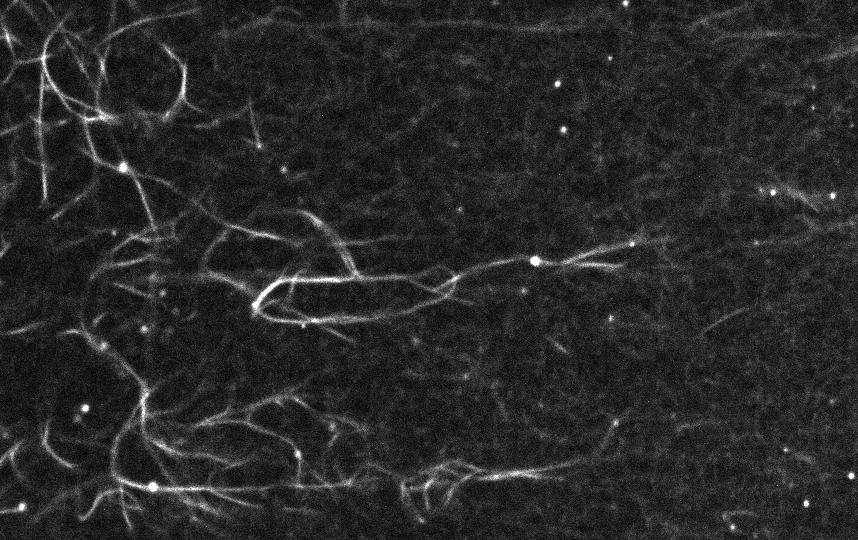

Supplement: Supplementary file 5 — Source data Fig. 4 [file 44319_2025_437_MOESM5_ESM.zip › Figure 4/Figure 4L/+200 nM Espin.tif]

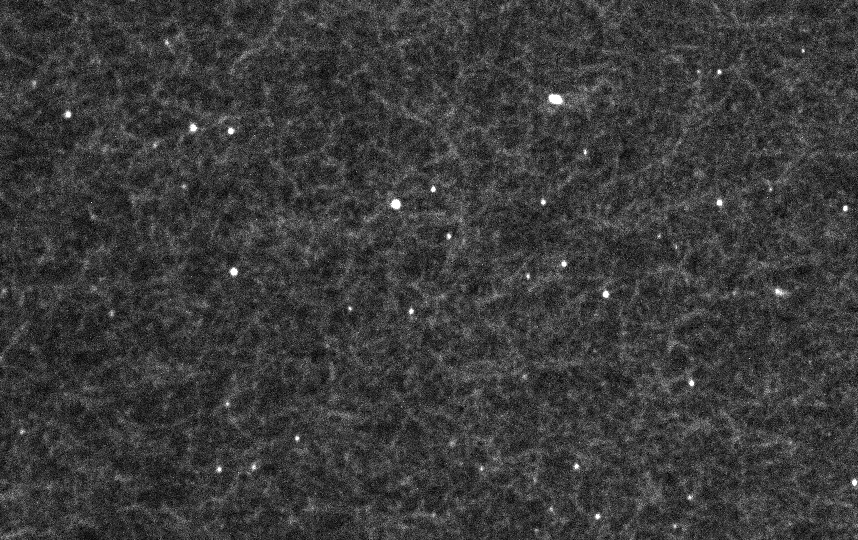

Supplement: Supplementary file 5 — Source data Fig. 4 [file 44319_2025_437_MOESM5_ESM.zip › Figure 4/Figure 4L/+200 nM Fascin.tif]

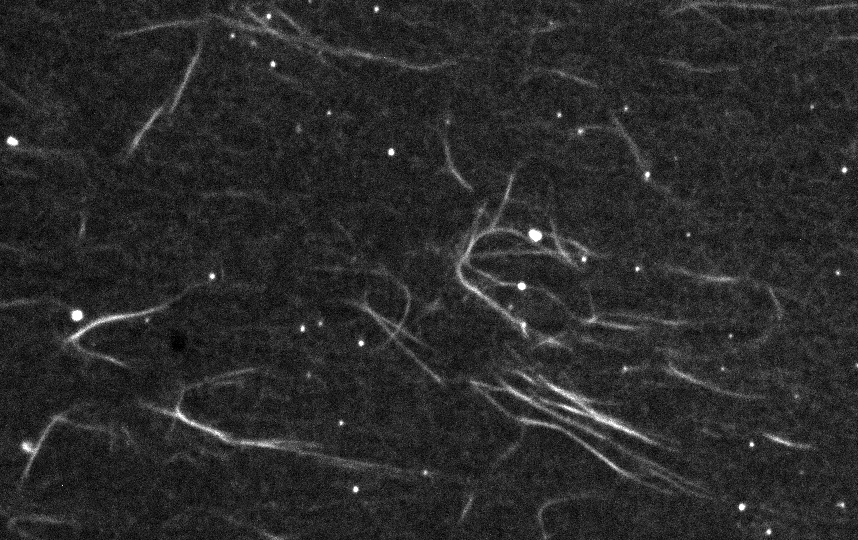

Supplement: Supplementary file 5 — Source data Fig. 4 [file 44319_2025_437_MOESM5_ESM.zip › Figure 4/Figure 4L/+500 nM Espin.tif]

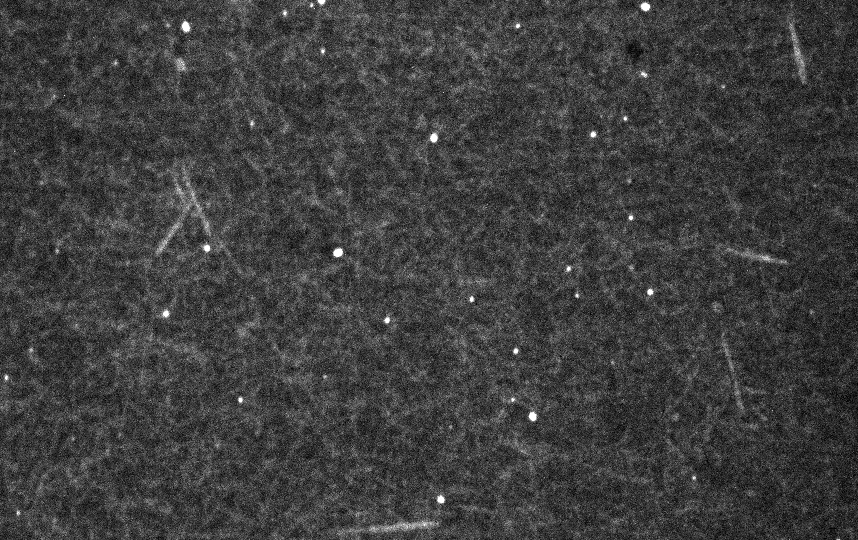

Supplement: Supplementary file 5 — Source data Fig. 4 [file 44319_2025_437_MOESM5_ESM.zip › Figure 4/Figure 4L/+500 nM Fascin.tif]

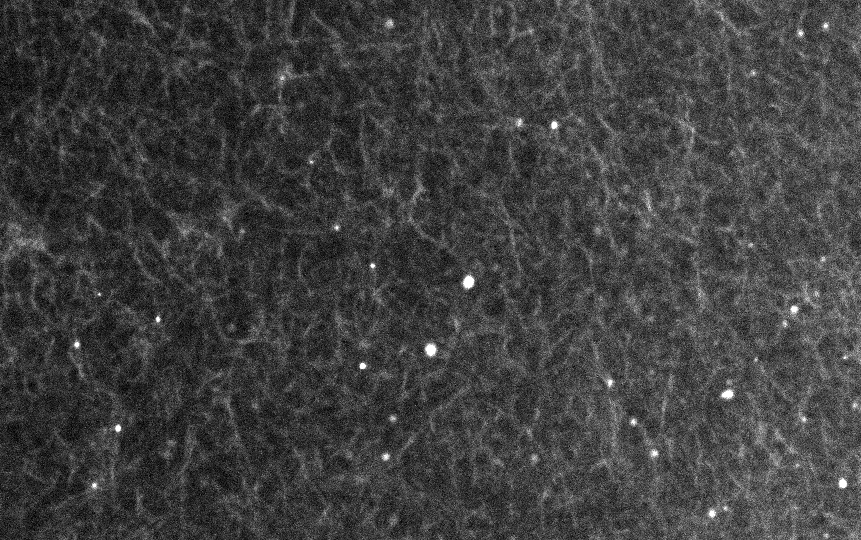

Supplement: Supplementary file 5 — Source data Fig. 4 [file 44319_2025_437_MOESM5_ESM.zip › Figure 4/Figure 4L/None.tif]

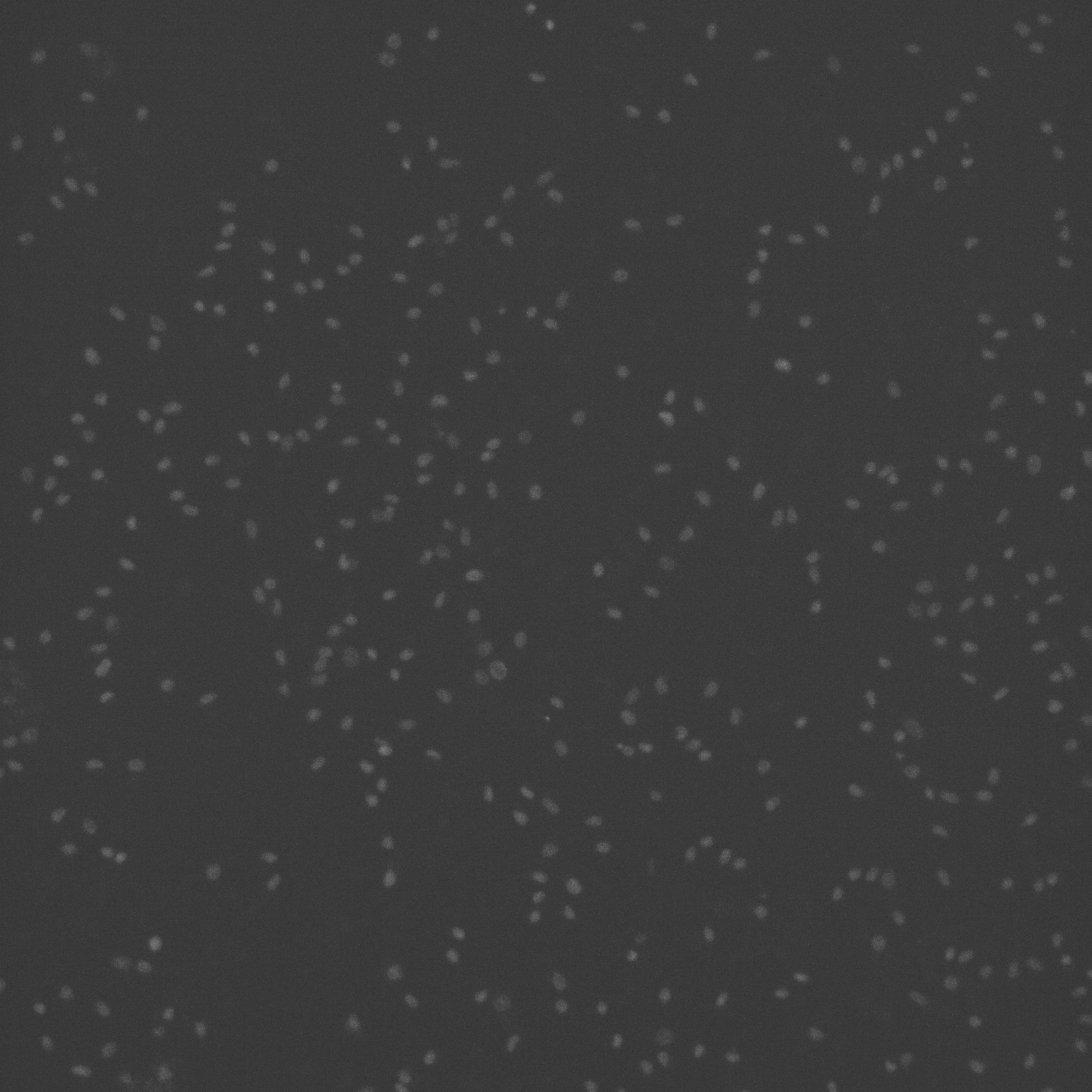

Supplement: Supplementary file 6 — Source data Fig. 5 [file 44319_2025_437_MOESM6_ESM.zip › Figure 5/Figure 5C/siCtrl.tif]

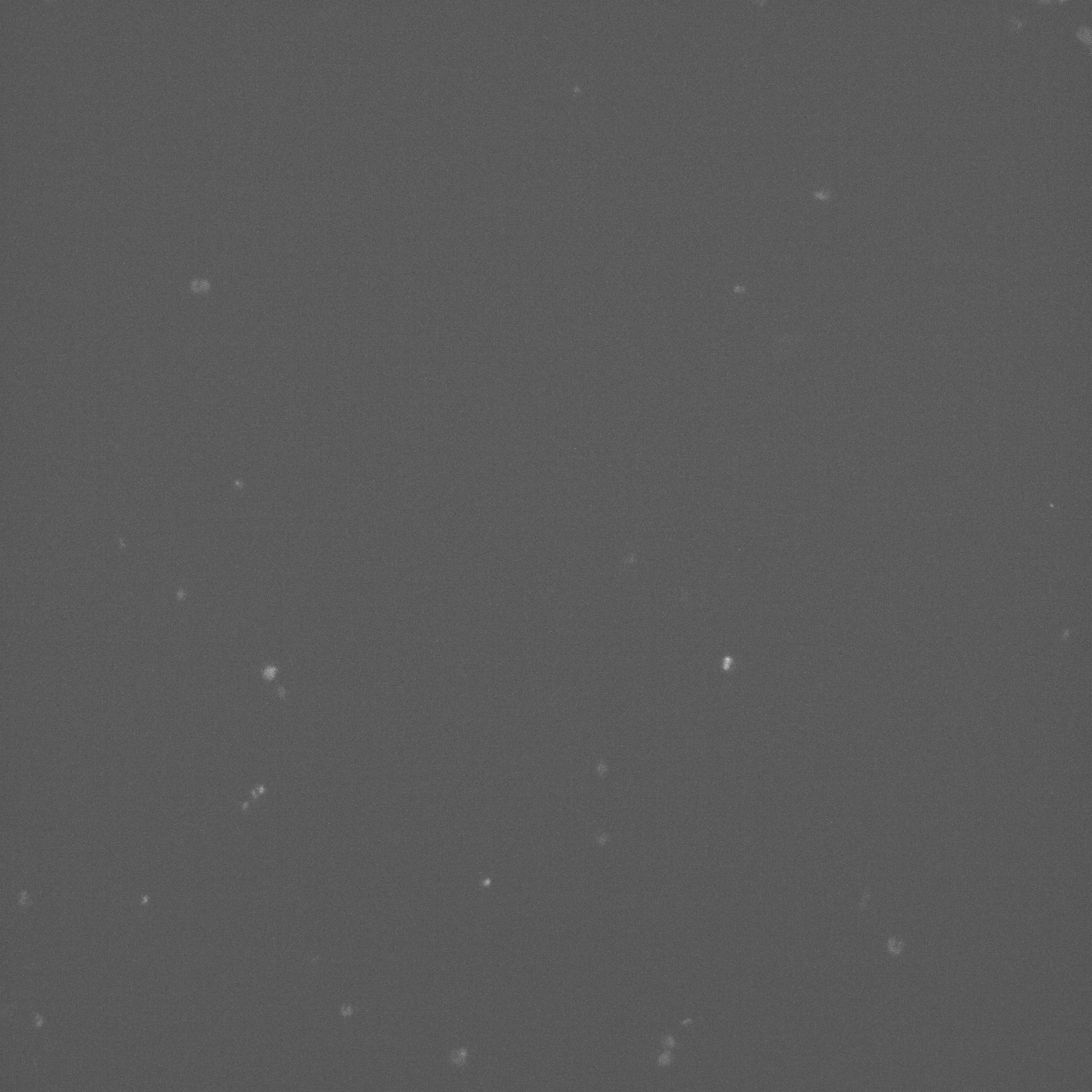

Supplement: Supplementary file 6 — Source data Fig. 5 [file 44319_2025_437_MOESM6_ESM.zip › Figure 5/Figure 5C/siFascin.tif]

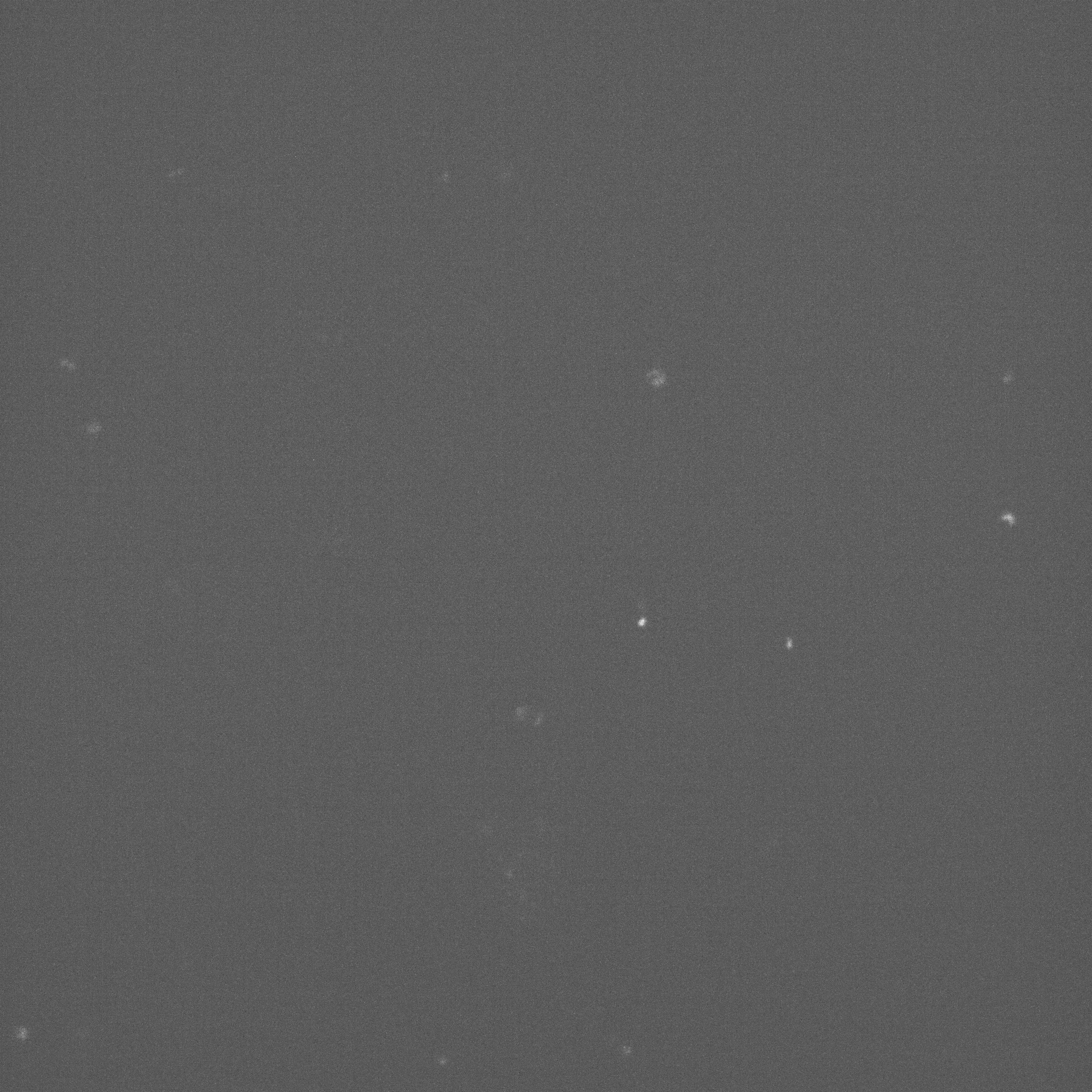

Supplement: Supplementary file 6 — Source data Fig. 5 [file 44319_2025_437_MOESM6_ESM.zip › Figure 5/Figure 5C/siMyosin-X.tif]

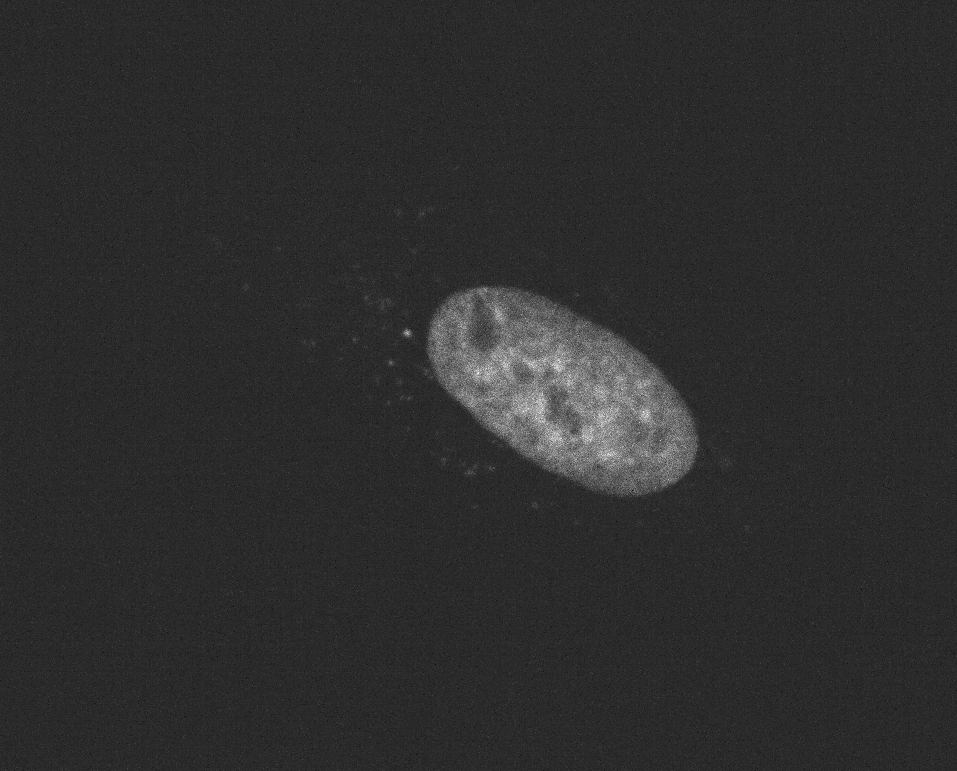

Supplement: Supplementary file 6 — Source data Fig. 5 [file 44319_2025_437_MOESM6_ESM.zip › Figure 5/Figure 5E/Ctrl.tif]

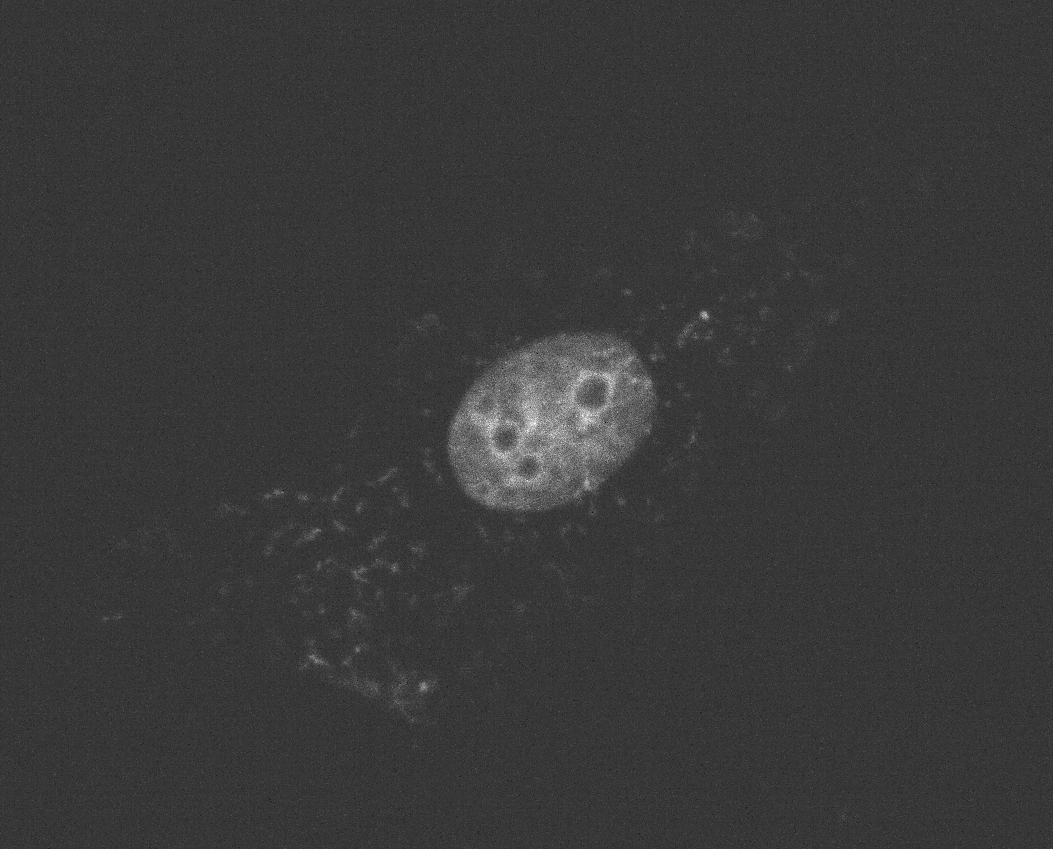

Supplement: Supplementary file 6 — Source data Fig. 5 [file 44319_2025_437_MOESM6_ESM.zip › Figure 5/Figure 5E/Espin OE.tif]

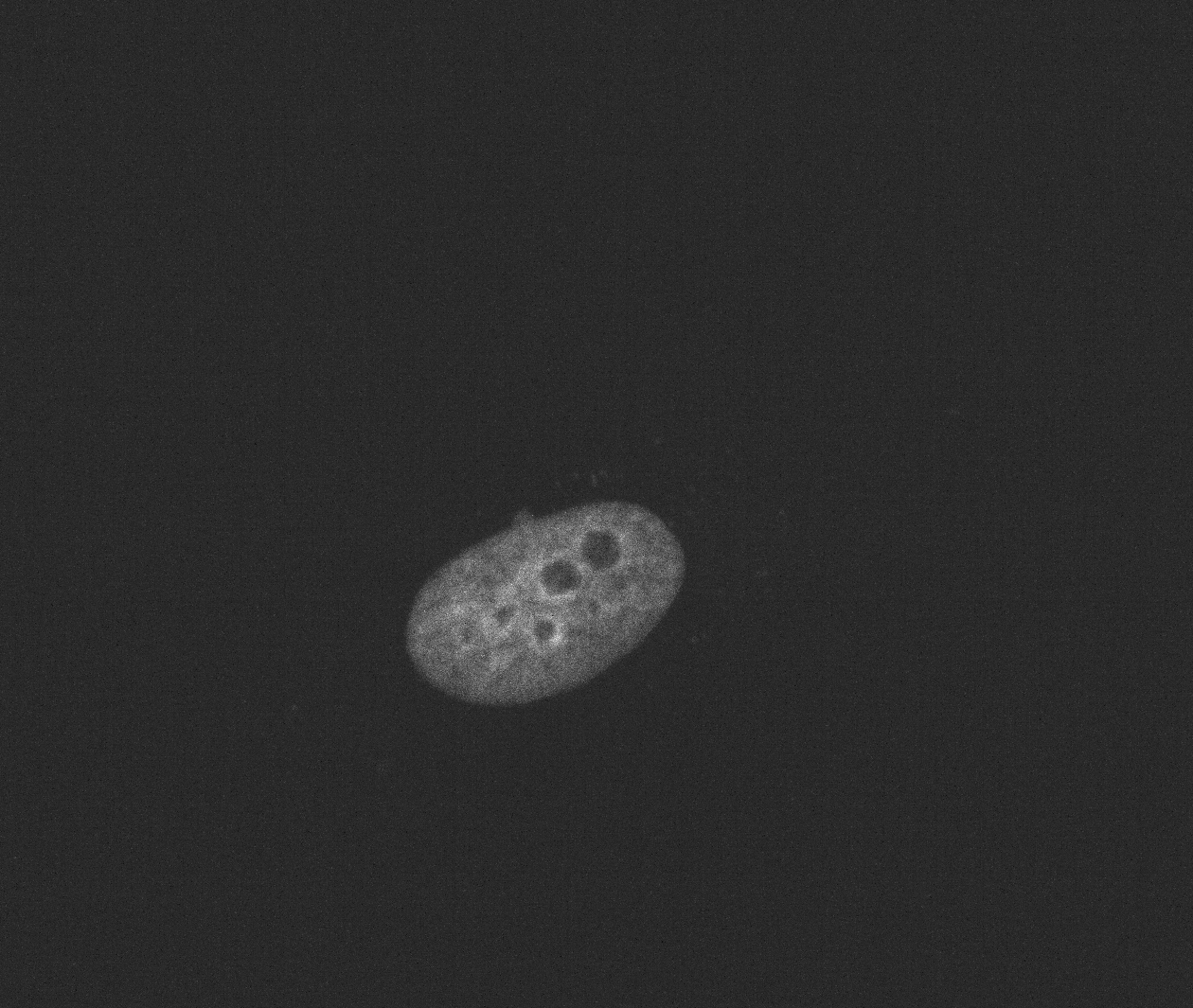

Supplement: Supplementary file 6 — Source data Fig. 5 [file 44319_2025_437_MOESM6_ESM.zip › Figure 5/Figure 5E/Espin ΔABM.tif]

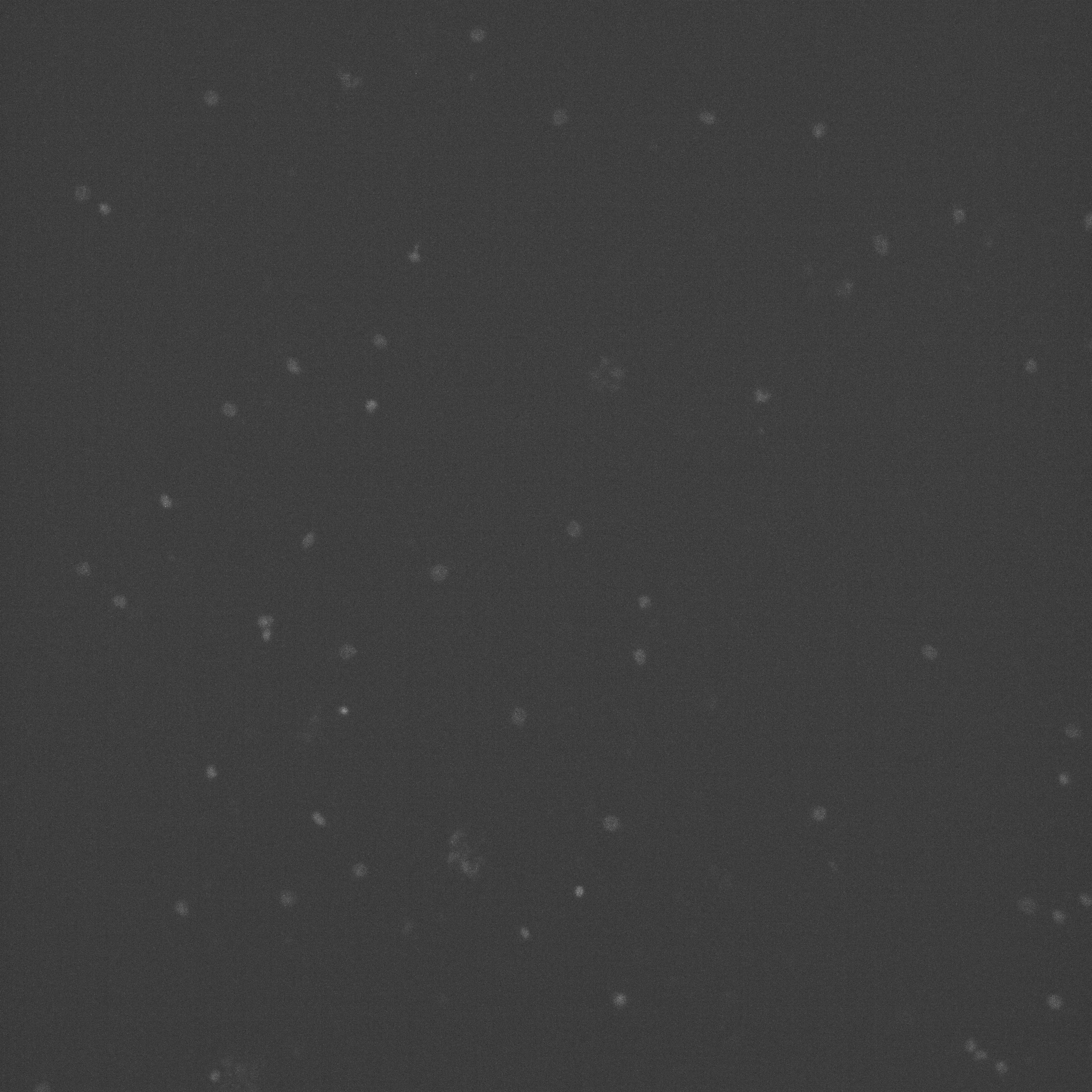

Supplement: Supplementary file 6 — Source data Fig. 5 [file 44319_2025_437_MOESM6_ESM.zip › Figure 5/Figure 5G/Ctrl.tif]

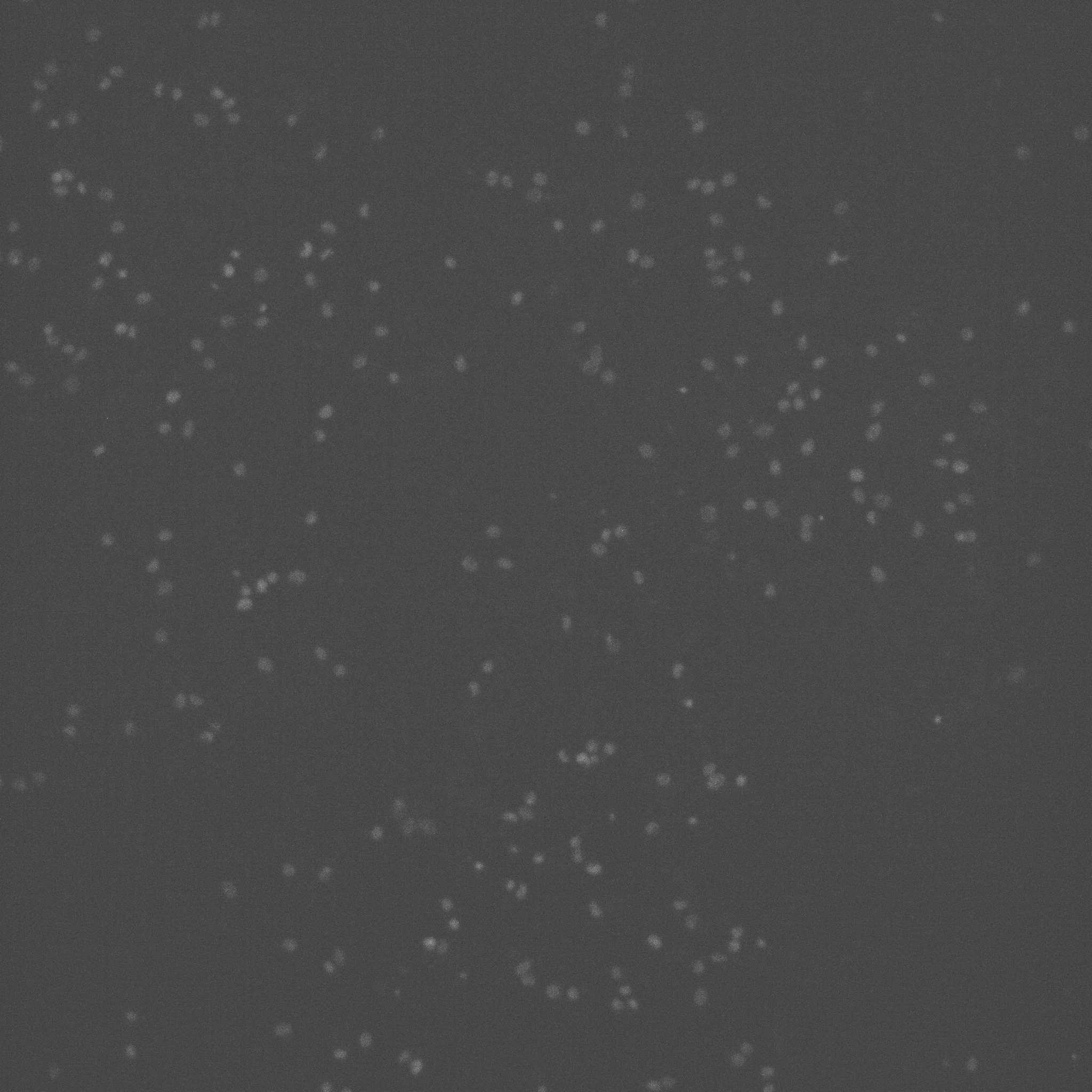

Supplement: Supplementary file 6 — Source data Fig. 5 [file 44319_2025_437_MOESM6_ESM.zip › Figure 5/Figure 5G/Espin OE.tif]

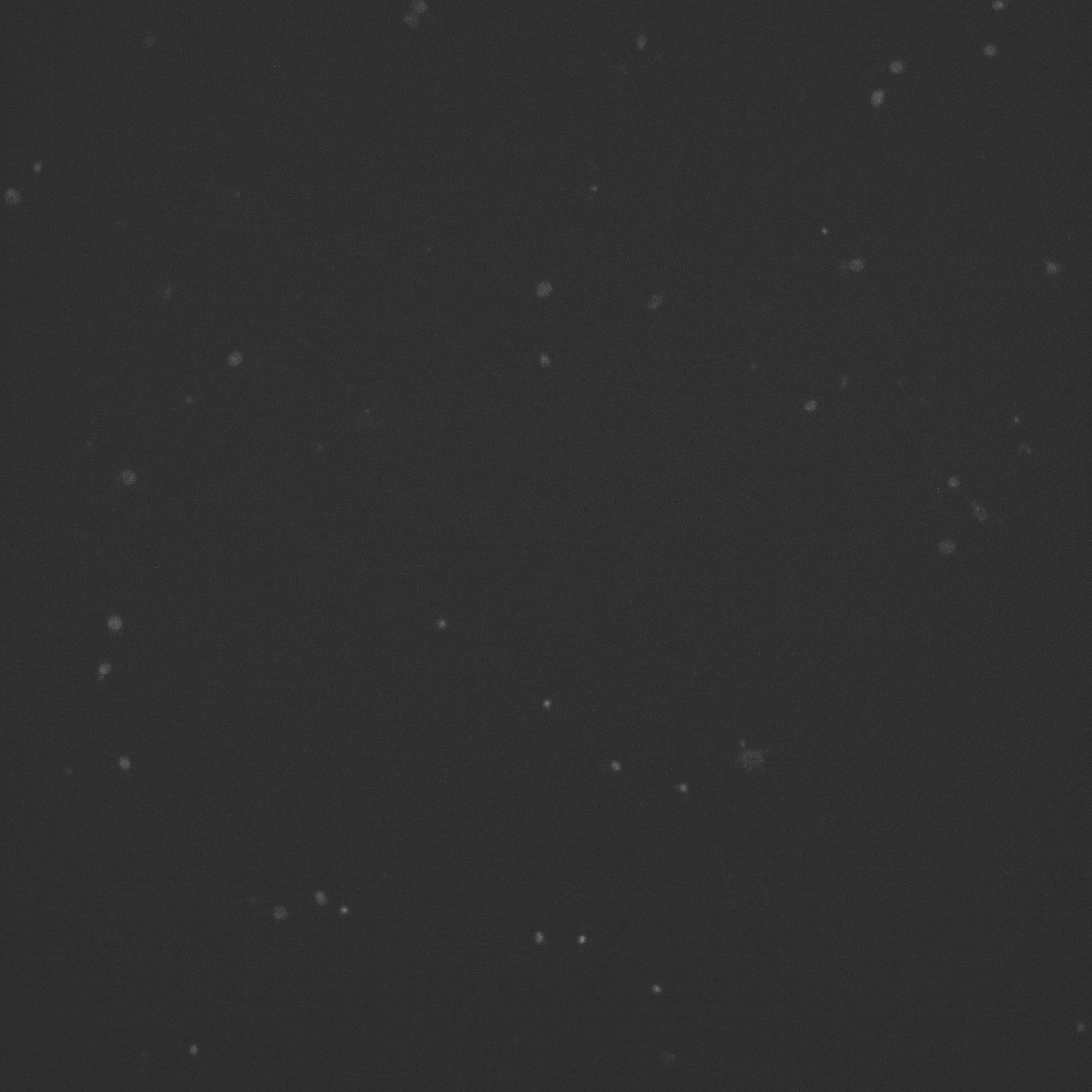

Supplement: Supplementary file 6 — Source data Fig. 5 [file 44319_2025_437_MOESM6_ESM.zip › Figure 5/Figure 5G/Espin ΔABM.tif]

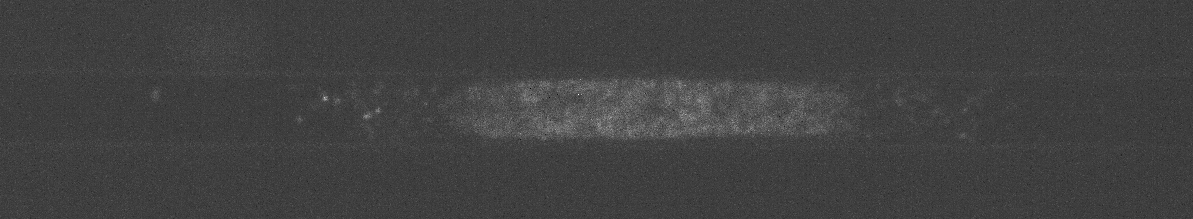

Supplement: Supplementary file 7 — Source data Fig. 6 [file 44319_2025_437_MOESM7_ESM.zip › Figure 6/Figure 6A/Ctrl.tif]

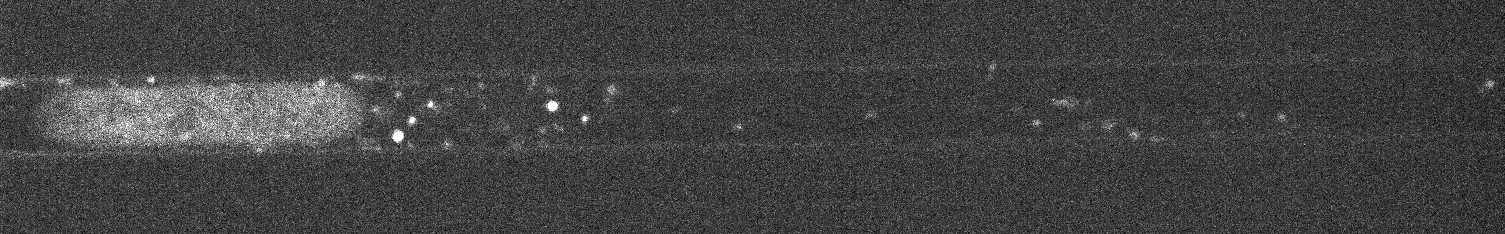

Supplement: Supplementary file 7 — Source data Fig. 6 [file 44319_2025_437_MOESM7_ESM.zip › Figure 6/Figure 6A/Espin OE.tif]

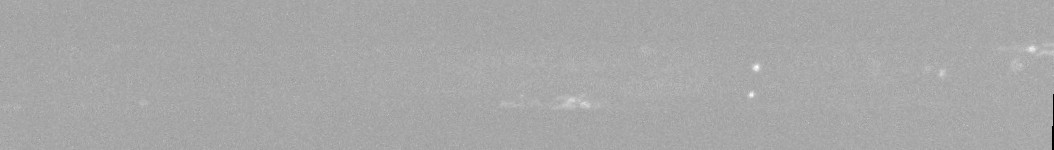

Supplement: Supplementary file 7 — Source data Fig. 6 [file 44319_2025_437_MOESM7_ESM.zip › Figure 6/Figure 6D/Figure 6D.tif]

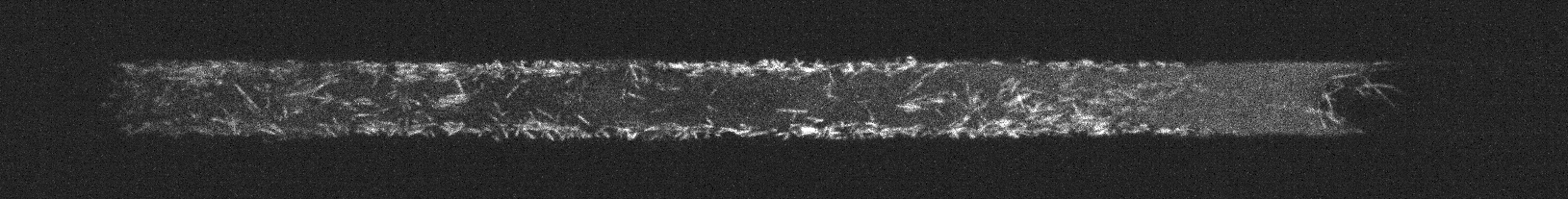

Supplement: Supplementary file 7 — Source data Fig. 6 [file 44319_2025_437_MOESM7_ESM.zip › Figure 6/Figure 6E/Bottom.tif]

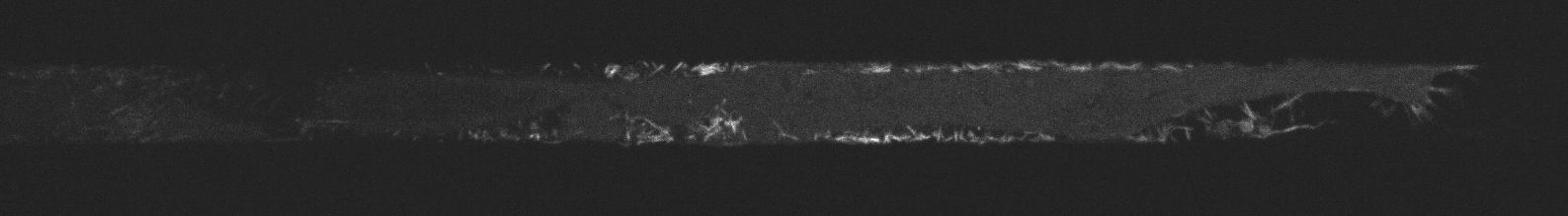

Supplement: Supplementary file 7 — Source data Fig. 6 [file 44319_2025_437_MOESM7_ESM.zip › Figure 6/Figure 6E/Side.tif]

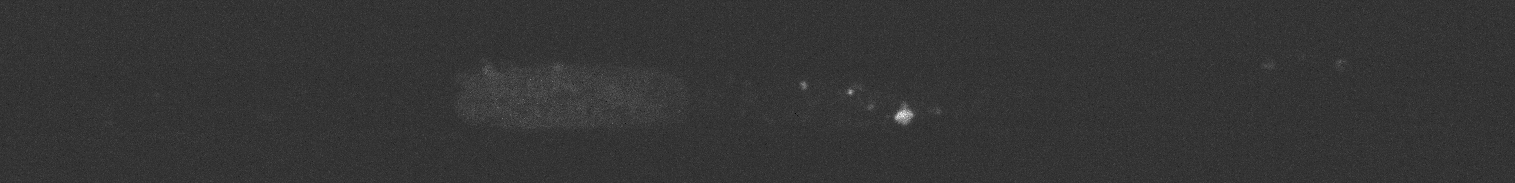

Supplement: Supplementary file 7 — Source data Fig. 6 [file 44319_2025_437_MOESM7_ESM.zip › Figure 6/Figure 6F/Figure 6F.tif]

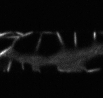

Supplement: Supplementary file 7 — Source data Fig. 6 [file 44319_2025_437_MOESM7_ESM.zip › Figure 6/Figure 6G/Bottom.tif]

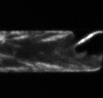

Supplement: Supplementary file 7 — Source data Fig. 6 [file 44319_2025_437_MOESM7_ESM.zip › Figure 6/Figure 6G/Upper.tif]
