# Supplementary material for: Espin enhances confined cell migration by promoting filopodia formation and contributes to cancer metastasis
Source: EMBO Rep. 2025 Apr 4;26(10):2574–96. doi: 10.1038/s44319-025-00437-1 (PMC12117036; doi:10.1038/s44319-025-00437-1)
Supplement: Supplementary file 8 — Expanded View Figures [file 44319_2025_437_MOESM8_ESM.pdf]

Expanded View Figures

Figure EV1. Related to Fig. 1.

(**A**) Confocal z-stacks taken with 1 µm steps show that the constriction length of transwell is approximately 10 µm. Stack 1 shows bottom nuclei on transwell while stack 12 shows upper nuclei. Dashed circles mark the representative bottom and upper nuclei. Scale bar: 20 µm. (**B**) Western blotting showing espin KD. (**C**) Cell growth curve of control and espin OE cells using CCK8 kit. Data represent technical replicates and are shown as mean ± SD. $n_{(Ctrl)} = 5$, $n_{(Espin\ OE)} = 5$. (**D**) Cell growth curve of control and espin KD cells. Data represent technical replicates and are shown as mean ± SD. $n_{(shCtrl)} = 5$, $n_{(shEspin\text{-}1)} = 5$, $n_{(shEspin\text{-}2)} = 5$. (**E**) Representative images showing control (just blue) and espin OE cells (cyan and blue) in microchannels. The nuclei were stained with Hoechst and displayed as blue. Scale bar: 20 µm. (**F**) Representative nuclear displacement of control and espin OE cells in confined microchannels with 5 µm width. The nuclei were stained with Hoechst and displayed as blue. Scale bar: 20 µm.

© The Author(s)

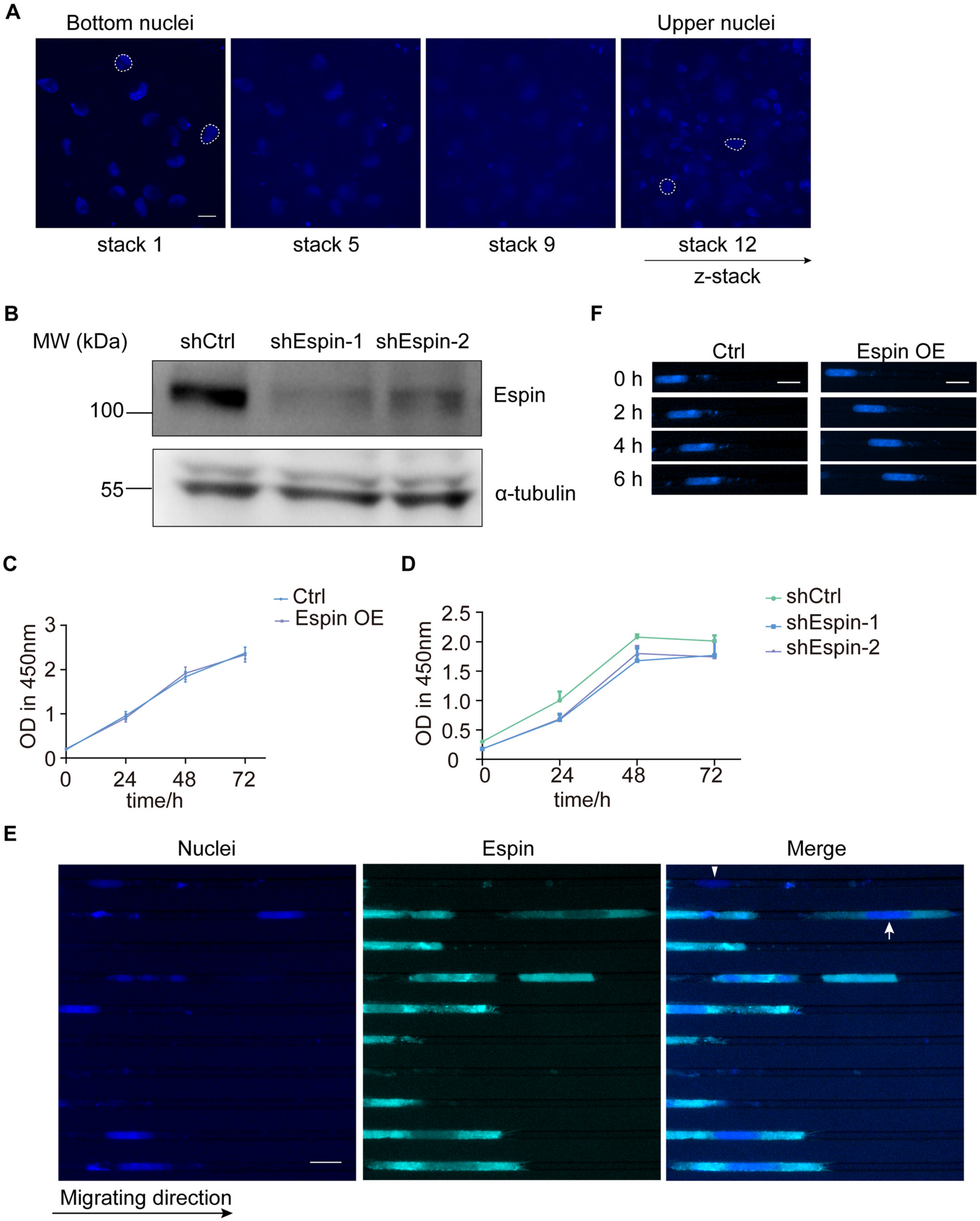
A
Bottom nuclei
Upper nuclei
stack 1
stack 5
stack 9
stack 12
z-stack
B
MW (kDa)
shCtrl
shEspin-1
shEspin-2
100
55
Espin
α-tubulin
F
Ctrl
Espin OE
0 h
2 h
4 h
6 h
C
Ctrl
Espin OE
OD in 450nm
time/h
D
shCtrl
shEspin-1
shEspin-2
OD in 450nm
time/h
E
Nuclei
Espin
Merge
Migrating direction

 © The Author(s)

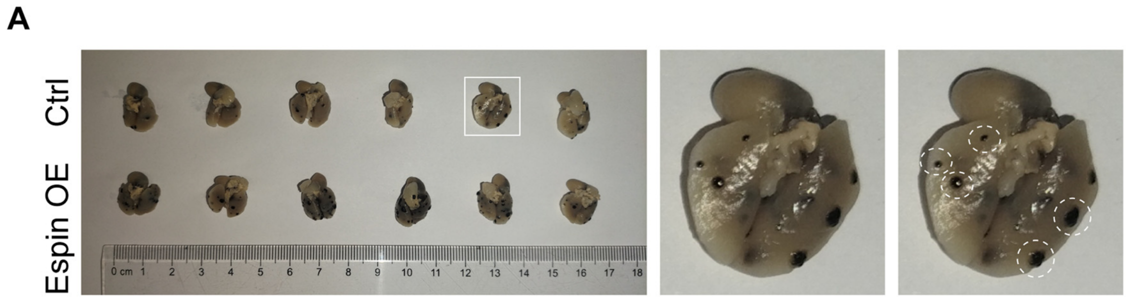

Figure EV2. Related to Fig. 2.

(**A**) Metastatic foci are morphologically circular and black to be visible on the lung surface, as shown in dashed circles.

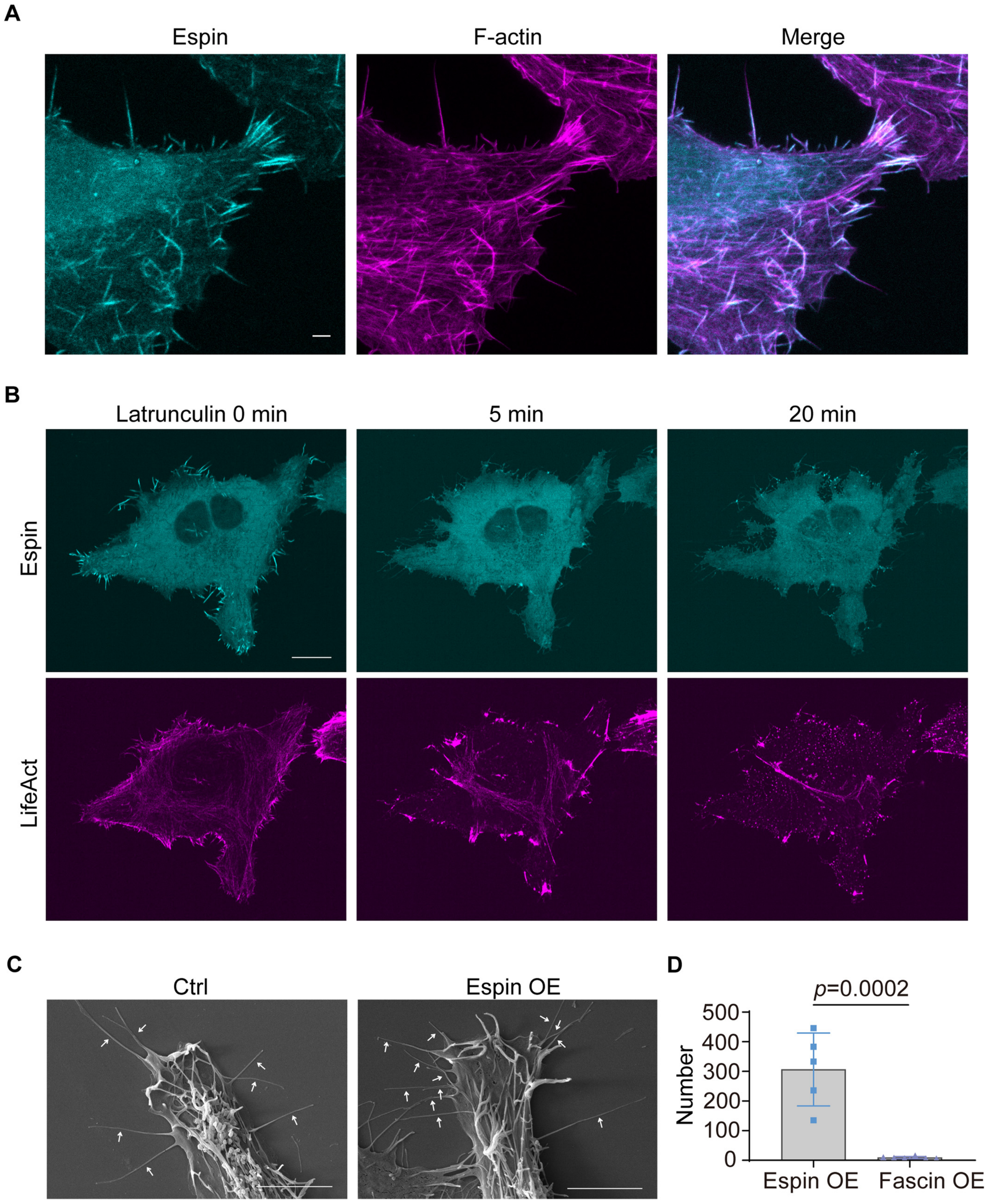
A
Espin
F-actin
Merge
B
Latrunculin 0 min
5 min
20 min
Espin
LifeAct
C
Ctrl
Espin OE
D
p=0.0002
500
400
300
200
100
0
Number
Espin OE
Fascin OE

 © The Author(s)

Figure EV3. Related to Fig. 4.

(**A**) Representative images of F-actin staining and espin-EGFP using STED. Scale bar: 5 µm. (**B**) Signals of LifeAct and espin. Cells transfected with espin and LifeAct were treated with 250 nM latrunculin B. Scale bar: 20 µm. (**C**) Representative images of cell morphology using SEM, triangles mark filopodia. Scale bar: 5 µm. (**D**) Quantification of dorsal filopodia number per cell in Fig. 4I. Data represent technical replicates and are shown as mean ± SD. $n_{(\text{Espin OE})} = 5$, $n_{(\text{Fascin OE})} = 6$. Significance was tested using unpaired Student's *t* test.

© The Author(s)

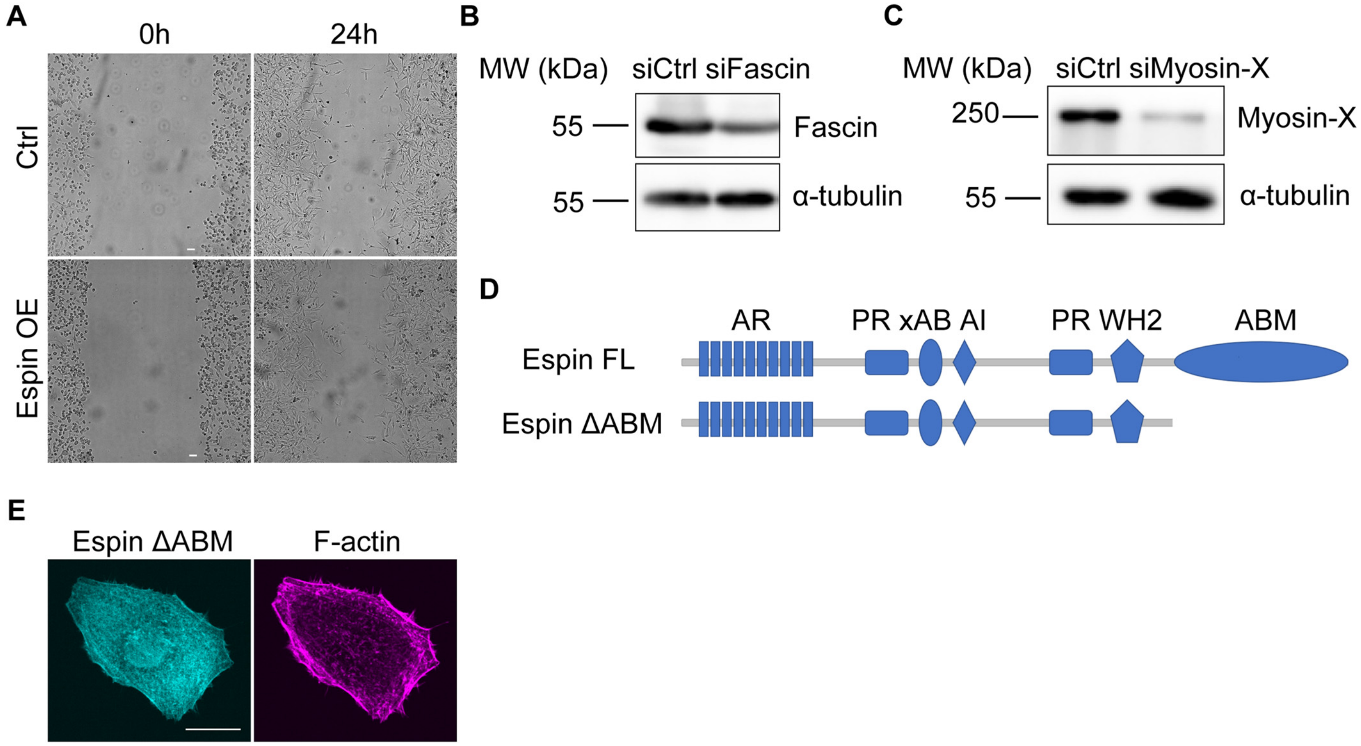

Figure EV4. Related to Fig. 5.

(**A**) Representative images showing the initial and 24 h area in wound healing assay. Scale bar: 50 μm. (**B**) Western blotting showing fascin KD in espin OE cells. Due to the similar molecular weights of fascin and α-tubulin, siCtrl and siFascin samples were loaded twice on the same 10% SDS-PAGE acrylamide gel. (**C**) Western blotting showing myosin-X KD in espin OE cells. (**D**) The structure of espin full length (FL) and the mutant depleting ABM. (**E**) Fluorescent signals of espin ΔABM and F-actin. Scale bar: 20 μm.

 © The Author(s)

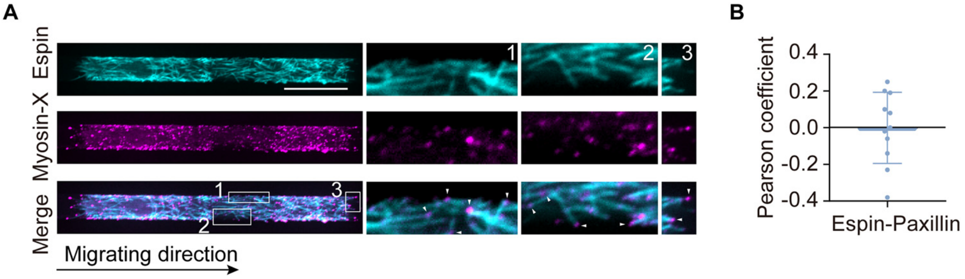

Figure EV5. Related to Fig. 6.

(**A**) Representative images of myosin-X localization and espin in confined channels. Espin OE cells were transfected with myosin-X. Cell areas in white boxes are enlarged. Triangles mark representative myosin-X at the tip of espin-protrusions. Scale bar: 20 µm. (**B**) The Pearson coefficient of espin and paxillin in confined cells. To filter unspecific background signals, a manual intensity threshold was used when using the Coloc2 in ImageJ to calculate Pearson coefficients. Data represent technical replicates and are shown as mean ± SD. $n_{(Espin\text{-}Paxillin)} = 11$.

© The Author(s)
